# Effects of Lactoferrin on Oral and Throat Conditions under Low Humidity Environments: A Randomized, Double-Blind, and Placebo-Controlled Crossover Trial

**DOI:** 10.3390/nu15184033

**Published:** 2023-09-18

**Authors:** Shutaro Kubo, Hirotsugu Oda, Miyuki Tanaka, Takashi Koikeda, Shinichi Tomita

**Affiliations:** 1 Innovative Research Institute, R&D Division, Morinaga Milk Industry Co., Ltd., 5-1-83 Higashihara, Zama 252-8583, Japan; 2 Shiba Palace Clinic, Daiwa A Hamamatsucho Bldg. 6F, 1-9-10 Hamamatsucho, Minato 105-0013, Japan; 3 Department of Advanced Food Sciences, Faculty of Agriculture, Tamagawa University, 6-1-1 Tamagawa-Gakuen, Machida 194-8610, Japan

**Keywords:** lactoferrin, low humidity, throat discomfort, oral discomfort, healthy adult

## Abstract

To evaluate the effects of a single ingestion of bovine lactoferrin (bLF) on oral and throat conditions under a low-humidity environment. A randomized, double-blind, 2-sequence, 2-treatment, and 2-period placebo-controlled crossover trial was conducted. Healthy adult subjects orally ingested bLF dissolved in water, or placebo water, followed by exposure to low humidity (20 °C, 20% relative humidity (RH)) for 2 h. The primary endpoint was subjective oral and throat discomfort assessed by a visual analog scale (VAS), which positively correlated with the discomfort. Secondary endpoints were unstimulated whole salivary flow rate (UWSFR) and salivary immunoglobulin A (IgA) secretion rate. Overall, 40 subjects were randomly assigned to two sequences (20 each) and 34 were analyzed. The VAS values for oral and throat discomfort in the bLF treatment were significantly lower than in the placebo treatment, whereas UWSFR and IgA secretion rates were comparable between the two treatments. Adverse drug reactions were not observed. Subjective oral and throat discomfort associated with low humidity is suppressed by a single ingestion of bLF. Our findings demonstrate the novel use of bLF in a clinical situation that leverages its unique characteristics.

## 1. Introduction

Lactoferrin (LF) is an iron-binding glycoprotein present in bodily fluids such as mammalian breast milk, saliva, tears, and nasal secretions, exhibiting various biological functions [1,2,3]. The quantity of saliva and its components are important for maintaining oral health. Saliva contains LF and other bioactive substances such as lactoperoxidase, immunoglobulin A (IgA), lysozyme, and mucin [4]. Among these substances, saliva constitutes approximately 8 µg/mL of LF [5] which plays an important role in maintaining oral hygiene via antimicrobial activity [6]. Bovine lactoferrin (bLF), isolated from cow milk, is used as a functional food ingredient [7] and oral administration of bLF is beneficial for maintaining oral hygiene [8] as endogenous lactoferrin is. In addition, a recent study indicated bLF and mucin form a complex with water molecules displaying high lubricity. This intricate arrangement holds the potential to improve crucial oral functions such as swallowing and speech through decreasing oral friction [9].

The amount of moisture present in the mouth is determined by the equilibrium between salivation and saliva evaporation. Loss of moisture in the mouth due to reduced salivation and/or excessive saliva evaporation can cause xerostomia [10]. Patients with xerostomia complain of oral and throat symptoms, such as dryness and speech and swallowing difficulties [11,12,13]. In a low-humidity environment, even healthy people experience such oral and throat discomfort transiently [14,15,16]. In Japan, indoor air frequently becomes dry during winter and office workers often complain of transient discomfort that negatively affects their quality of life, such as oral and throat discomfort [16]. A possible cause of this discomfort is derived from a reduction in moisture in the mouth and throat due to excessive saliva evaporation caused by low humidity [17]. However, limited approaches exist, such as the ingestion of functional food or supplements to counteract moisture reduction easily and effectively. Based on the in vitro study that mucin and bLF form a complex with water molecules [9], we expected that this complex can be also formed in the mouth and retain moisture on oral and throat mucosa. Therefore, we hypothesized that intake of bLF would alleviate transient dryness in the mouth and throat attributed to reduced humidity levels. Therefore, we conducted a clinical trial to evaluate the effects of bLF ingestion under low-humidity conditions that simulate the Japanese winter indoor environment. However, there is no objective method for evaluating aspects of oral and throat conditions (e.g., throat dryness). Therefore, we evaluated subjective oral and throat discomfort due to low humidity using a visual analog scale (VAS). In addition, we assessed the unstimulated whole salivary flow rate (UWSFR), which reflects the amount of oral moisture, to explore the underlying mechanisms. The salivary IgA secretion rate, which is associated with mucosal immunity, was also assessed to explore the benefits of maintaining saliva quantity.

## 2. Materials and Methods

### 2.1. Ethical Approval

This study was conducted in accordance with the current version of the Declaration of Helsinki [18] and the ethical guidelines for medical and health research involving human participants [19]. The study protocol was approved by the Ethics Committee of Tamagawa University on 1 September 2022 (approval no. TRE22-0024) and registered with the UMIN Clinical Trials Registry (UMIN000048878). Written informed consent was obtained from all participants.

### 2.2. Study Design

This randomized, double-blind, two-sequence, two-treatment, and two-period placebo-controlled crossover trial was conducted at Tamagawa University (Tokyo, Japan) between September and October 2022. The alleviation of oral and throat discomforts was expected to be derived from the direct effects of bLF, mucin, and water complexes on oral and throat mucosa. Therefore, we considered that carryover effects were negligible and chose a crossover design with a 1-week washout period. The test had four periods: recruitment, assessment of eligibility, and randomization (1 month); treatment period 1 (1 day); washout (1 week); and treatment period 2 (1 day). The participants were randomly assigned (1:1) to ingest bLF in treatment period 1, followed by a placebo in treatment period 2 (Sequence A), or placebo in treatment period 1, followed by bLF in treatment period 2 (Sequence B). The intervention was conducted once during each treatment period.

### 2.3. Participants

The participants were healthy adults from Tamagawa University. The inclusion criteria was being aged between 18–65 years. The exclusion criteria were as follows: (1) salivary flow rate less than 0.1 mL/min (diagnostic criteria for xerostomia); (2) disease in the teeth, mouth, pharynx, larynx, esophagus, or respiratory system; (3) consumption of functional foods or use of oral care products that influence the oral environment during the test period; (4) mouth breather; (5) burden on the mouth, pharynx, larynx, esophagus, and respiratory system; (6) smoker; (7) heavy drinker; (8) irregular wake-up time and bedtime; (9) serious disease in the liver, kidney, heart, lung, gastrointestinal tract, blood, endocrine system, and metabolic system; chronic disease; or serious medical history of these; (10) serious food allergy, such as milk or drug allergies, or a serious medical history of these conditions; (11) pregnant, under lactation, or expected to be pregnant during the course of the study; and (12) determined as ineligible for participation in this study, judging from the principal researcher/doctor’s opinions on the findings of their background. The background information confirmed by the principal researcher/doctor included recent hospital records, medication status, subjective symptoms, and basic demographics such as sex, age, body weight, and height. In determining the sample size, guided by an estimation of α error = 0.05, β error = 0.8, δ = 14.0, and σ = 30.0, the sample size was computed as 39 using EZR (version 1.40) leading us to set the target sample size as 40, accounting for the logistical aspects of the experimental procedure.

### 2.4. Randomization and Blinding

The participants were randomly allocated to sequence A or B by a third-party allocation manager. A randomization list was generated using a stratified method with sex as the stratified factor. The original randomization list was sealed until the key code was broken. The allocation manager or their substitute prepared the test foods. The test foods were bLF and the placebo. The bLF was 100 mg of MLF-1^®^ (Morinaga Milk Industry, Tokyo, Japan), cow’s milk-derived bLF concentrate, and it contained 96.3 mg of bLF (96.3% purity). bLF was dissolved in water before oral ingestion. We conducted a preliminary test to determine the optimal dose at which the effects of bLF could be expected and found that bLF was still retained in saliva at 28.8 and 1.3 µg/mL 1 and 2 h, respectively, after a single ingestion of 100 mg of MLF-1^®^. Therefore, we set the dose as above. The placebo was water used to dissolve MLF-1^®^. Prepared test foods were introduced into opaque bottles to make them indistinguishable. Then, the allocation manager or their substitute wrote the participants’ numbers on the bottle according to the copy of the randomization list. Moreover, the allocation manager confirmed that the test foods were indistinguishable in appearance, taste, and smell. The trial staff provided the packages to the participants based on the assigned numbers. All the staff in this trial, besides the allocation manager or their substitute, were blinded until the key code was broken.

### 2.5. Intervention

**Figure 1:**
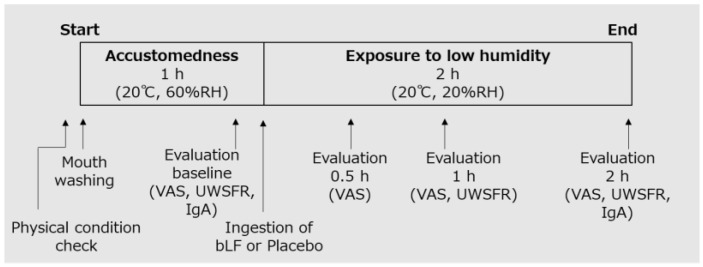
Intervention procedure. RH, relative humidity; VAS, visual analog scale; UWSFR, unstimulated whole salivary flow rate; bLF, bovine lactoferrin; IgA, immunoglobulin A.

The participants fasted for 2 h before the start of the treatment periods. Before commencement, a thorough health assessment was conducted on the participants, ensuring the absence of any subjective symptoms affecting the oral and throat regions, as well as the absence of fever (<37.5 °C). Following this confirmation, the participants drank water to wash their mouths and spent an hour within a general hydrothermal environment (20 °C, 60% relative humidity (RH)) to acclimatize. Subsequently, baseline evaluations were performed. Then, they orally ingested the test food and were exposed to low humidity (20 °C, 20% RH) representative of the indoor environment during winter in Japan [14,16]. They remained in the environment for 2 h and were evaluated over time (Figure 1). During the test, the participants were prohibited from speaking or wearing masks.

### 2.6. Endpoints

The primary endpoints were subjective oral and throat discomfort due to low humidity. Total oral and throat discomforts were assessed using the VAS, as described in previous studies [20,21]. Specific oral and throat discomforts (difficulty in speaking due to dryness, difficulty in swallowing due to dryness, amount of saliva in the mouth, dryness of the mouth, dryness of the throat, dryness of the lips, dryness of the tongue, and level of thirst) were assessed using the VAS, which is generally used for the diagnosis of xerostomia [22]. The VAS employed in this study consisted of a 100 mm straight horizontal line, with the rightmost end defined as extreme discomfort whereas the leftmost end was defined as extreme comfort. Participants were instructed to demarcate their perceived intensity of discomfort by marking the line at the corresponding point. The quantification of discomfort intensity was subsequently achieved by measuring the distance (mm) from the mark to the leftmost end of the line. The VAS was evaluated four times (baseline and after exposure to low humidity for 0.5, 1, and 2 h). The secondary endpoints were the UWSFR and salivary IgA secretion rate. The UWSFR was assessed for 5 min using the spitting method, as described in a previous study [23]. The UWSFR was evaluated thrice (baseline and after exposure to low humidity for 1 and 2 h). The IgA secretion rate was calculated from the UWSFR and IgA concentrations in the saliva collected for the evaluation of the UWSFR. The IgA concentration was assessed at two time points (baseline and after exposure to low humidity for 2 h). The IgA concentration in the saliva was quantified at the Yanaihara Institute (Shizuoka, Japan) using an enzyme-linked immunosorbent assay (ELISA).

### 2.7. Statistical Analysis

Statistical analyses were conducted per the protocol set. The primary and secondary endpoints, VAS value of discomfort, UWSFR, and salivary IgA secretion rate were analyzed using a linear mixed model in which the treatment, period, and baseline values were fixed effects and participant identity was a variable effect. The analysis was conducted in accordance with a closed testing procedure to avoid multiple comparisons. The carryover effect was analyzed using a model in which the interaction between treatment and time was added to the fixed effects of the main analysis model. The differences between the treatments at baseline and between each time point and baseline for each treatment were analyzed using a paired *t*-test. Differences in the frequency of adverse events among the treatments were evaluated using the McNemar test. Demographic data were analyzed using the chi-square test for nominal variables and the *t*-test for continuous variables. The correlation between the change in the UWSFR and the VAS score was analyzed using Pearson’s correlation coefficient. Statistical analysis was performed using JMP version 14.0 (SAS Institute Inc., Cary, NC, USA) and the level of statistical significance was set at *p* < 0.05 (carryover effect *p*-value < 0.10).

## 3. Results

### 3.1. Participants

**Figure 2:**
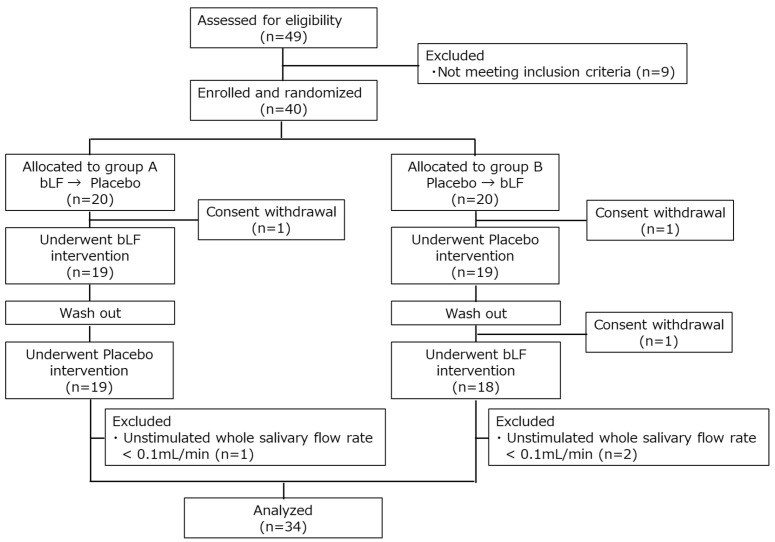
CONSORT flow diagram. bLF, bovine lactoferrin.

The participants were recruited from 7 to 22 September 2022. In total, 40 healthy adults were enrolled; 20 were assigned to sequence A (bLF first) and the others were assigned to sequence B (placebo first). Overall, 3 participants dropped out due to consent withdrawal and 37 completed the test. In total, 3 other participants were excluded for meeting the exclusion criteria (UWSFR < 0.1 mL/min), leaving 34 for the per-protocol analysis (Figure 2). The demographic information regarding the participants in this study is presented in Table 1.

### 3.2. Primary Endpoint

Regarding the comprehensive assessment of oral and throat discomforts, the VAS scores for total oral discomfort were significantly higher at the 1–2 h period for both the placebo and bLF treatments when compared with the baseline. However, a significant reduction in VAS scores was observed at 2 h for the bLF treatment, contrasting with the placebo treatment. Similarly, the VAS scores for total throat discomfort were significantly elevated at the 1–2 h period in the placebo and at the 2 h juncture in the bLF treatment compared to baseline levels. Again, VAS scores were significantly lower at 2 h for the bLF treatment when compared to the placebo treatment (Table 2). No carryover effects were detected (*p* > 0.10). Regarding specific oral and throat discomforts, the VAS score at the end of the test denoting “Difficulty in swallowing due to dryness”, “Dryness of the throat”, “Dryness of the lips”, and “Level of thirst” were significantly lower in the bLF treatment group than in the placebo treatment group (Table 3).

### 3.3. Secondary Endpoints

The UWSFR was significantly higher at 2 h for the placebo and at 1–2 h for the bLF treatment compared with baseline levels, though there were no significant differences observed between groups (Table 4). The salivary IgA secretion rate remained unaltered from the baseline levels following each treatment and there were no significant differences evident following each treatment across various time points (Table 5).

### 3.4. Exploratory Analysis

The variation in the UWSFR (0–1 h) was negatively correlated with the VAS scores at 2 h regarding total oral and throat discomfort (Table S1). 

### 3.5. Adverse Events

Side effects or severe adverse events were evaluated in all the participants who ingested the test food. There was one adverse event, namely abdominal pain, after placebo treatment (1/37) and no adverse event after bLF treatment (0/36). No significant difference was observed between the two treatments. The principal doctor denied a causal relationship between ingesting the test food and the adverse event.

## 4. Discussion

In a low-humidity environment, oral and throat discomfort increased with time and a single ingestion of bLF before exposure to the environment yielded a notable alleviation of the discomfort in comparison to the placebo. Although the UWSFR following bLF treatment consistently demonstrated higher values across each time point in contrast to the placebo, statistical analysis did not reveal any significant disparity between the two treatments. In addition, it is worth noting that the augmentation in UWSFR was more pronounced at earlier time points subsequent to bLF treatment than after the placebo treatment. 

For the inclusion of participants, a physician conducted a comprehensive assessment, ensuring their overall health status. For those with a UWSFR less than 0.1 mL/min, the diagnostic criteria for xerostomia were excluded [24]. Moreover, prior to exposure to the low humidity conditions (20 °C, 60% RH), the participants did not experience any subjective symptoms or discomfort. The experimental low humidity conditions (20 °C, 20% RH) were driven by considerations of an indoor environment reminiscent of the winter season in Japan [14,16]. Therefore, the transitory discomfort experienced by participants was not attributed to underlying ailments such as xerostomia or the common cold. Instead, it was directly linked to the low-humidity environment, a scenario encountered in routine daily life. Consistent with previous studies [15], the healthy participants in this study experienced environmental discomfort after exposure to low humidity, regardless of bLF or placebo ingestion. 

In this study, we employed the VAS, which was initially developed for diagnosing xerostomia, to meticulously assess distinct instances of oral and throat discomfort. It is worth noting this VAS has been validated and found to exhibit a negative correlation with salivary flow rates [22]. Additionally, the VAS utilized to gauge total oral and throat discomfort demonstrated a comparable negative correlation with the UWSFR (Table S1). This phenomenon strongly suggested a tangible influence of salivary volume within the mouth on the discomfort evaluations measured by the VAS. Results of the VAS underscored the efficacy of bLF treatment in ameliorating both total oral and throat discomfort due to low humidity. All VAS values regarding specific oral and throat discomfort were also lower in the bLF treatment group than in placebo treatment but a significant difference was not observed in part of them probably due to the small sample size. In addition, the impact of bLF on specific oral and throat discomfort varied among different sites. Notably, bLF treatment effectively alleviated specific discomforts such as “Difficulty in swallowing due to dryness”, “Dryness of the throat”, “Dryness of the lips”, and “level of thirst”; however, it did not exhibit any alleviation of discomfort manifestations like “Difficulty in speaking”, “Amount of saliva in the mouth”, “Dryness of the mouth”, and “Dryness of the tongue”. This pattern suggests that the effect of bLF is more distinct in throat mucosa compared to mouth mucosa, which could be attributed to the anatomical and physiological differences between these regions. The mouth mucosa surfaces, with the exception of the upper jaw, are perpetually coated by secreted saliva, mitigating the impact of low humidity-induced drying. On the contrary, the throat’s mucosa, located distally from salivary glands, lacks a consistent wetting mechanism unless activated by swallowing. Consequently, under conditions of low humidity, the drying throat mucosa supersedes the dampening effect of saliva, potentially accentuating the impact of variations in saliva volume or moisturizing efficacy. This discrepancy in moisture distribution and drying dynamics within the mouth and throat mucosa could account for the observed divergence in VAS scores, despite the gradual increase in UWSFR increase over time. Additionally, the humidity of inhaled air holds significance for the proper functioning of the trachea. High-humidity conditions are conducive to enhancing the ciliary-generated flow within the trachea [25,26], facilitating the expulsion of foreign substances. It is plausible that bLF ingestion engenders an increase in moisture within the throat mucosa. This moisture increment could contribute to the increased humidity during inhalation, thereby fostering an enhanced clearance of foreign particles by the cilia lining the trachea, and may explain the observed alleviation of throat discomfort following bLF treatment observed in this study. 

The cause of discomfort due to the low-humidity environment was most likely owing to the lack of moisture in the mouth, similar to xerostomia. Therefore, the mechanism by which bLF suppresses the discomfort may involve the maintenance of moisture in the mouth. Our findings suggest three possible mechanisms involved in this maintenance. The first revolves around the possibility of increasing the salivary flow rate. The UWSFR at each time point was higher following the bLF treatment compared to the placebo treatment, albeit without achieving statistical significance. In addition, UWSFR significantly increased over shorter time intervals subsequent to bLF treatment, as opposed to the placebo treatment. These facts support the possibility of an increased UWSFR following bLF treatment. Psychological tension decreases salivation [27] and a previous clinical study suggested that tension distorts the measurement of the salivary flow rate [28]. In this study, some participants confessed tension regarding the unfamiliar experimental environment at the baseline evaluation and gradual adjustment to the environment. In fact, the baseline UWSFR was lower than that after exposure to low humidity, even with placebo treatment (Table 4). Therefore, it is conceivable that the baseline value was possibly lower than the true value due to the tension experienced by some of the participants. As the degree of psychological tension differed among participants, this may have caused measurement variability in the UWSFR. By accounting for the influence of psychological tension, it becomes feasible to potentially enhance the clarity of bLF’s effects on the UWSFR. The second reason is the suppression of saliva evaporation. In our preliminary experiment, the bLF solution was added to the saliva sample (final concentration of bLF 30 µg/mL) and it was dropped onto a weight pan, followed by air drying for 2 h. As a result, the weight decrease was notably lower when compared to that of saliva with added water. The final concentration of bLF in the saliva sample (30 µg/mL) was approximately equivalent to the concentration 1 h after bLF ingestion in the current clinical trial. This suggests that bLF helps to maintain moisture in the mouth by suppressing saliva evaporation. The third reason is the improvement in salivary wettability. Saliva with added bLF solution dropped onto an acrylic resin showed a lower contact angle on the plate compared to that with added water. The contact angle is an indicator of wettability; the lower the contact angle, the higher the wettability. Highly wettable saliva is expected to spread evenly on the mucosal membrane and maintain surface moisture. 

The salivary IgA secretion rate is associated with mucosal immunity and protection against foreign substances. Our expectation was that under the conditions of a low humidity environment, alongside the UWSFR, the secretion rate would also decrease. Furthermore, we postulated that bLF administration might mitigate this decline by maintaining the salivary output. However, our observations revealed that these rates remained comparable to those observed in the placebo treatment group. 

The conclusions drawn from this study point towards a direct influence of bLF on oral and throat mucosa. Consequently, the mechanisms underpinning bLF’s effects are expected to be operational regardless of its delivery form, encompassing various consumable formats such as chewable and liquid products, except for completely insoluble food forms, such as enteric-coated products. In our pilot test, the concentration of bLF in saliva decreased below a few µg/mL in 2 h after its ingestion. Thus, even low concentrations of bLF may effectively suppress oral and throat discomfort caused by low humidity.

In this study, we found that the ingestion of bLF suppressed subjective oral and throat discomfort due to a low-humidity environment. In addition, we discussed the possibility that bLF functions via the following mechanisms: modulating the UWSFR, saliva evaporation, and salivary wettability. However, this study had some limitations. Firstly, this method to assess discomfort is subjective because no objective method is generally used for the assessment. Therefore, the results of the subjective evaluation may have been subject to bias. Secondly, the experimental environment itself engendered physiological tension in some participants, potentially influencing the measurement outcomes. Thirdly, we did not evaluate the effects of other bLF-containing food forms than aqueous bLF solution. And finally, we could not measure the bLF concentration in the saliva after ingestion of bLF to confirm whether ingested bLF remained in the mouth. In our forthcoming studies, we intend to address these limitations and conduct a clinical trial to obtain more reliable results. Intake of bLF is beneficial for oral hygiene due to its antibacterial activity. However, knowledge regarding the other uses of bLF for oral health is limited. Based on our findings, we propose a novel use of bLF for oral and throat health that alleviates oral and throat discomfort in drying conditions as well as the possibility of its use as in easily consumable food products.

## Figures and Tables

**Table 1:** Baseline demographics.

	All Participants	Sequence A	Sequence B	*p*-Value
Participants, *n*	34	18	16	
Sex (F/M), *n*	18/16	10/8	8/8	0.746
Age, years (SD)	22.6 (6.1)	23.0 (6.4)	22.1 (5.7)	0.668
Body weight, kg (SD)	55.9 (11.1)	55.5 (10.8)	56.3 (11.3)	0.849
Height, m (SD)	1.65 (0.083)	1.64 (0.095)	1.65 (0.069)	0.887

SD, standard deviation.

**Table 2:** VAS values regarding total oral and throat discomfort due to low humidity.

Outcome	Period of Exposure	Placebo mm, Mean (SE)	bLF mm, Mean (SE)	*p*-Value ^1^	*p*-Value ^2^	*p*-Value ^3^
Oral discomfort	Baseline (0 h)	15.4 (2.2)	14.1 (2.4)	0.590	-	-
	0.5 h	15.5 (1.7)	15.1 (2.3)	0.668	0.957	0.373
	1 h	19.8 (1.9)	16.6 (2.2)	0.126	0.026	0.029
	2 h	23.2 (2.2)	17.6 (2.2)	0.027	0.003	0.011
Throat discomfort	Baseline (0 h)	16.9 (2.2)	17.8 (3.6)	0.764	-	-
	0.5 h	18.9 (1.9)	18.3 (3.1)	0.345	0.311	0.829
	1 h	24.0 (2.5)	21.3 (3.3)	0.101	0.008	0.141
	2 h	30.7 (3.4)	23.9 (3.3)	0.002	0.001	0.028

^1^: placebo vs. bLF at each time point; ^2^: baseline vs. each time point following placebo treatment; ^3^: baseline vs. each time point following bLF treatment; bLF, bovine lactoferrin; SE, standard error SE.

**Table 3:** VAS values regarding specific oral and throat discomfort due to low humidity.

Outcome	Period of Exposure	Placebo mm, Mean (SE)	bLF mm, Mean (SE)	*p*-Value ^1^	*p*-Value ^2^	*p*-Value ^3^
Difficulty in speaking due to dryness	Baseline (0 h)	14.9 (2.5)	13.1 (2.3)	0.373	-	-
	0.5 h	15.5 (2.0)	13.0 (2.1)	0.249	0.657	0.914
	1 h	17.8 (2.1)	14.5 (2.2)	0.297	0.157	0.192
	2 h	20.5 (2.6)	16.2 (2.4)	0.248	0.046	0.054
Difficulty in swallowing due to dryness	Baseline (0 h)	15.7 (2.9)	15.2 (2.8)	0.883	-	-
	0.5 h	16.0 (2.3)	15.4 (2.6)	0.868	0.799	0.833
	1 h	20.9 (2.7)	17.3 (2.9)	0.149	0.011	0.056
	2 h	24.5 (3.1)	19.0 (3.1)	0.042	0.001	0.019
Amount of saliva in the mouth	Baseline (0 h)	32.6 (3.4)	33.5 (3.6)	0.748	-	-
	0.5 h	32.4 (3.0)	32.1 (3.5)	0.545	0.879	0.279
	1 h	37.0 (3.1)	33.9 (3.4)	0.106	0.041	0.853
	2 h	39.4 (3.3)	35.6 (3.5)	0.129	0.017	0.317
Dryness of the mouth	Baseline (0 h)	16.4 (2.2)	17.2 (2.7)	0.754	-	-
	0.5 h	16.9 (1.9)	17.9 (2.7)	0.749	0.733	0.623
	1 h	20.0 (1.8)	20.6 (2.8)	0.894	0.077	0.085
	2 h	25.6 (2.6)	22.3 (2.9)	0.129	0.001	0.033
Dryness of the throat	Baseline (0 h)	18.4 (2.2)	19.9 (3.3)	0.592	-	-
	0.5 h	19.7 (1.7)	19.6 (2.9)	0.404	0.553	0.891
	1 h	25.5 (2.4)	23.2 (3.3)	0.163	0.016	0.193
	2 h	31.9 (3.4)	26.0 (3.5)	0.012	0.001	0.035
Dryness of the lips	Baseline (0 h)	35.1 (3.4)	32.5 (3.9)	0.544	-	-
	0.5 h	41.4 (3.2)	38.3 (3.8)	0.274	<0.001	0.001
	1 h	48.0 (3.3)	42.6 (3.9)	0.021	<0.001	<0.001
	2 h	54.0 (3.5)	46.0 (4.2)	0.009	<0.001	<0.001
Dryness of the tongue	Baseline (0 h)	18.4 (2.7)	18.1 (3.1)	0.945	-	-
	0.5 h	17.7 (2.3)	17.7 (2.8)	0.788	0.653	0.744
	1 h	22.0 (2.8)	19.8 (2.8)	0.404	0.091	0.242
	2 h	25.3 (3.2)	21.6 (3.0)	0.226	0.019	0.047
Level of thirst	Baseline (0 h)	27.5 (3.1)	30.6 (3.5)	0.289	-	-
	0.5 h	28.4 (2.8)	29.6 (3.0)	0.559	0.700	0.573
	1 h	36.6 (3.2)	33.3 (3.3)	0.037	0.001	0.228
	2 h	42.7 (3.4)	37.2 (3.6)	0.013	<0.001	0.023

^1^: placebo vs. bLF at each time point; ^2^: baseline vs. each time point following placebo treatment; ^3^: baseline vs. each time point following bLF treatment; bLF, bovine lactoferrin; SE, standard error.

**Table 4:** Unstimulated whole salivary flow rate.

Outcome	Period of Exposure	Placebo µL/min, Mean (SE)	bLF µL/min, Mean (SE)	*p*-Value ^1^	*p*-Value ^2^	*p*-Value ^3^
Unstimulated whole salivary flow rate (UWSFR)	Baseline (0 h)	532.9 (46.4)	526.8 (50.2)	0.826	-	-
	1 h	558.0 (46.3)	577.4 (49.6)	0.349	0.207	0.037
	2 h	581.7 (52.2)	610.5 (57.6)	0.269	0.049	0.003

^1^: placebo vs. bLF at each time point; ^2^: baseline vs. each time point following placebo treatment; ^3^: baseline vs. each time point following bLF treatment; bLF, bovine lactoferrin; SE, standard error.

**Table 5:** Salivary IgA secretion rate.

Outcome	Period of Exposure	Placebo µg/min, Mean (SE)	bLF µg/min, Mean (SE)	*p*-Value ^1^	*p*-Value ^2^	*p*-Value ^3^
IgA secretion rate	Baseline (0 h)	200.1 (15.4)	210.0 (15.5)	0.396	-	-
	2 h	202.1 (14.7)	211.3 (14.9)	0.865	0.866	0.905

^1^: placebo vs. bLF at each time point; ^2^: baseline vs. each time point following placebo treatment; ^3^: baseline vs. each time point following bLF treatment; bLF, bovine lactoferrin; SE, standard error.

## Data Availability

The data that support the findings of this study are available from the corresponding author upon reasonable request.

## Supplementary Materials

The following supporting information can be downloaded at: https://www.mdpi.com/article/10.3390/nu15184033/s1, Table S1: Correlation between the change in UWSFR (Δ0–1 h) and the VAS value (2 h).
