# Supplementary material for: Effects of Lactoferrin on Oral and Throat Conditions under Low Humidity Environments: A Randomized, Double-Blind, and Placebo-Controlled Crossover Trial
Source: Nutrients. 2023 Sep 18;15(18):4033. doi: 10.3390/nu15184033 (PMC10537525; doi:10.3390/nu15184033)
Supplement: Supplementary file 1 [file nutrients-15-04033-s001.zip › nutrients-2601650-supplementary.pdf]

Table S1. Correration between the change in UWSFR (Δ0-1 h) and VAS value (2 h)

**Outcome (VAS)**	**r**	***P*-value**
Oral discomfort	-0.277	0.022
Throat discomfort	-0.284	0.019

r: correlation coefficient
